# Factors associated with unsuccessful treatment outcome in tuberculosis patients among refugees and their surrounding communities in Gambella Regional State, Ethiopia

**DOI:** 10.1371/journal.pone.0205468

**Published:** 2018-10-18

**Authors:** Eyasu Ejeta, Getenet Beyene, Getu Balay, Zegeye Bonsa, Gemeda Abebe

**Affiliations:** 1 Mycobacteriology Research Center, Jimma University, Jimma, Ethiopia; 2 Department of Medical Laboratory Sciences and Pathology, Faculty Health Sciences, Jimma University, Jimma, Ethiopia; 3 Department of Medical Laboratory Sciences, College of Medical and Health Sciences, Wollega University, Nekemte, Ethiopia; Indian Institute of Technology Delhi, INDIA

## Abstract

**Background:**

Tuberculosis (TB) is a leading cause of public health challenges among immigrant refugees and their surrounding communities in developing countries. Evaluating the treatment outcome of TB patients is one of the key indicators to understand the performance of TB control program. Hence, this study aims to assess profile, treatment outcome and factors associated with unsuccessful outcome of TB patients treated under the TB control program among refugees and their surrounding communities (SCs) in Gambella Regional State, Ethiopia.

**Methodology:**

Retrospective study was conducted in the health facilities of refugee and their SCs in Gambella Regional State from March 1 to May 30, 2017. Demographic and related data of all TB patients registered in TB Control Program between September, 2008 and October, 2017 in health facilities of refugee and the SCs was extracted using data extraction format. Eight years trend of TB, treatment outcome and factors associated with unsuccessful outcome among refugees and the SCs were computed using SPSS version 20.0 software.

**Result:**

A total of 886 refugees and 3284 SCs TB patients, registered for anti TB treatment in the last eight years, were evaluated in the study. The trend of all forms of TB is progressively increasing among refugees contrary to the SCs in the course of the study period (X^2^ trend = 207.7; P<0.0001). Smear positive pulmonary TB (PTB+) was found to be predominant (57.6%) TB form in refugees while smear negative pulmonary TB (PTB-) (44.8%) is in the SCs (X^2^ = 185.834; P<0.0001). There was also significant difference in the treatment outcome (X^2^ = 170.915; P<0.0001). Mean treatment success rate was 74.2% and 88.1% for refugees and the SCs, respectively (X^2^ = 92.887; P<0.0001). The study also revealed that the risk of unsuccessful TB treatment outcome was significantly higher among refugee (AOR = 2.17; 95% CI: 1.69–2.77), retreated cases (AOR = 1.53; 95% CI: 1.07–2.17), patients aged between 35–44 years (AOR = 1.38; 95% CI: 1.0–1.91), and greater than 44 years old (AOR = 1.77; 95% CI: 1.28–2.44), and patients with extra pulmonary TB (EPTB) form (AOR = 1.34; 95% CI: 1.04–1.73) compared to their counterparts. Patient coming from rural area (AOR = 0.77; 95% CI: 0.62–0.97), who are female (AOR = 0.76; 95% CI: 0.63–0.91) and TB/HIV non-infected (AOR = 0.63; 95% CI: 0.51–0.77) were more likely to be successfully treated.

**Conclusion:**

The study confirmed that there was low treatment success rate among refugees compared to the SCs. Being refugee, retreated case, patient’s age ≥35 years old, EPTB form, gender, rural patient address and HIV status were predictor factors for unsuccessful treatment outcome. Hence, the study urges the need for strengthened TB prevention program among refugees with due consideration of identified predictor factors to prevent the potential effect of hosting refugee to the SCs and the nations at large.

## Introduction

Despite the availability of effective diagnosis tool and treatment, TB remains the top cause of death as a single infectious disease. It is caused by bacillus *Mycobacterium tuberculosis*. Around one third of the global population is infected. In 2017, 10.0 million people fell sick with TB and 1.3 million died of it [1]. More than 80% of the global TB burden and TB-related deaths were reported from high TB burden countries [1].

TB is a disease of poverty and it affects mostly socio-economically disadvantaged segments of population such as refugees. Refugees are exceptionally vulnerable to TB infection because of their low socio-economic status, living in overcrowded conditions, poor nutrition, and lack of access to health care services [2–5]. Although global plan of end TB programme is to achieve at least 90% of TB treatment coverage and to cure at least 90% of them in order to reduce TB transmission in the family and community [1], refugee TB control is challenged with difficulties to ensure adherence of patients to full course of treatment period and most (85%) of refugees originate from, and live in countries with high TB burden [6, 7].

Ethiopia has been hosting one of the largest refugees in Africa [8]. This is mainly due to the fact that the country is bordered by the most volatile and conflict ridden countries (South Sudan, Somalia, Eritrea) in the region [9]. It is one of the top 10 among the high TB burden countries [1]. The country is also among the high drug resistance TB burden and TB/HIV co-infected countries [1]. Directly Observed Treatment Short-term (DOTS) program was started in Ethiopia since 1992 [10]. Although there are recognizable variations from region to region and district to district in Ethiopia, the DOTS strategy has been subsequently scaled up and has reached 100% geographical and 95% institutional level coverage in the country [11]. The treatment and control of TB among refugees residing in Ethiopia relies on DOTS strategy as an important indicator to monitor and evaluate the case notification and treatment success as a measure of effectiveness of the program [12]. However, there is limited evidence on the trend of TB, treatment outcome and factors associated with unsuccessful treatment outcome among refugees and the SCs in the country. Hence, this study was conducted in Gambella Regional State residing refugees and surrounding communities health facilities providing TB treatment under national TB Control Program.

## Methodology

### Study setting

This study was conducted in three refugee camps (Jawi Refugee Health Center, Pugnido I and II refugee health Centers) and four SC health facilities (Gambella Hospital, Bonga Health Center, Gambella Health Center and Pugnido Health Center) where DOTS services have been provided for the last eight years. Each DOTS centers in refugee camps and the SCs follow the National TB Control Program Guideline for diagnosis, treatment and control of TB [10]. The Regional State Health Department is responsible for monitoring and evaluating the TB treatment program in both the refugee camps and the SCs. The Gambella Regional State hosts the largest (43.3%) number of refugees in Ethiopia [13]. The region has been hosting and providing humanitarian protection for a large number of refugees for the last three decades. Most of the refugees residing in the Gambella Regional State were come from South Sudan. The number of refugees residing in the region was alarmingly increasing following South Sudanese political instability since December 2013. In spite of introduction of the DOTS program in the region for more than decades, the region is still in the lead with TB and HIV burden compared to the national level.

### Study design and period

A retrospective study was conducted from March 1 to May 30, 2017 on all TB patients registered from September, 2008 to October, 2017 at health facilities providing DOTS services for refugees and the SCs in Gambella Regional State, Ethiopia.

### Data collection

**Fig 1:**
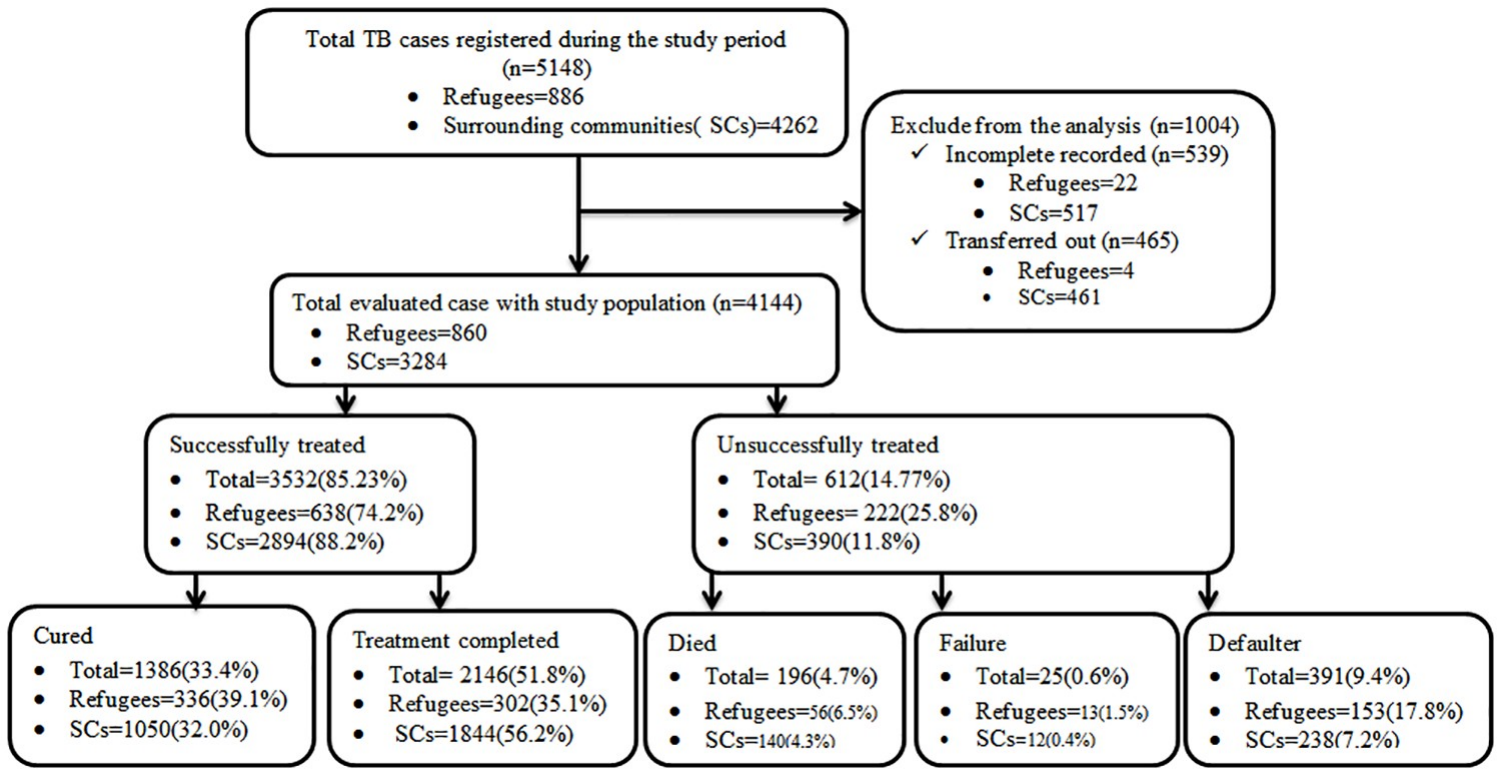
Number registered TB patients and treatment outcomes among refugees and surrounding communities during September, 2009- October, 2017 in Gambella Regional State, South West Ethiopia. Note: TB, Tuberculosis; SCs, surrounding communities. There were difference in the treatment outcome (X2 = 170.915; P<0.0001) and success rate (X2 = 92.887; P<0.0001) between the two study populations.

Tuberculosis treatment records in the study health facilities were used as the data source. The data were extracted using pre-developed data extraction format prepared for this purpose S1 Table. The collected data includes address of patients, sex, age, patient category, smear result, TB form, HIV status, treatment outcomes, and year of treatment. The study excluded TB patients with missed or unrecorded treatment outcomes and patients transferred to other health facilities, as information on their treatment outcome was not available. A total of 539 TB patients with incomplete record in treatment outcome and 465 patients transferred to other health facilities were excluded from the analysis (Fig 1).

### Definitions

According to National TB Control Program Guideline adopted from WHO, the following standard clinical case definition and treatment outcome rate operational terms were used [10]:

- **Smear-positive pulmonary TB (PTB+):** A patient diagnosed with at least two sputum smear microscopy positive for acid fast bacilli (AFB) or light-emitting diode (LED), or a patient with only one sputum smear microscopy positive for AFB or LED, and chest radiographic abnormalities consistent with active PTB.
- **Smear-negative pulmonary TB (PTB-):** A patient presented with symptom of TB, with at least two sputum smear microscopy and was negative for AFB or LED, or a patient with GeneXpert MTB detected and with chest radiological abnormalities consistent with active pulmonary TB and no response to two weeks of broad spectrum antibiotic therapy; diagnosis by clinician’s decision to treat with a full course of anti-TB chemotherapy.
- **Extra pulmonary TB (EPTB).** TB involving organs other than the lungs, such as bone, joints, lymph nodes, abdomen, and genitourinary tract, the meninges and others. EPTB diagnosis is based on GeneXpert/ biochemical analyses or histo-pathological evidence from a biopsy of affected organ with strong clinical evidence consistent with active EPTB by a clinician decision to treat with a full course of anti-TB chemotherapy.

According to National TB Control Program and WHO, treatment outcomes rate was categorized into:

- **Cure rate (CR):** The rate of a TB patient with initially sputum smear-positive who was converted to sputum smear-negative in the last month of treatment and on at least one previous occasion divided by the total smear positive TB cases in the same period.
- **Treatment success rate (TSR)**: A sum of PTB+ cases who were declared cured (patient with initially sputum smear-positive who was converted to sputum smear-negative in the last month of treatment and on at least one previous occasion) and, PTB- and EPTB cases who were declared treatment complete (a patient who completed treatment with resolution of symptoms, but who did not meet the criteria for cure or failure) divided by the total TB cases in the same period.
- **Unsuccessful treatment outcome rate:** The sum of patients with treatment result of failure (a patient who remains smear positive after a 5 month treatment period), defaulter (Patients who interrupted treatment for 2 consecutive months or more after commencing the treatment) and patients who died of any reason during the course of treatment divided by the total TB cases in the same period.

### Data management and analysis

Completeness of the data was checked visually and coded, entered, cleaned and analyzed using IBM SPSS for window version 20.0 statistical software. Descriptive statistics (frequency, proportion, mean and standard deviation) were used to summarize patients’ characteristics across the outcome variables. The independent predicators of unsuccessful treatment outcomes were presented using crude odds ratios (CORs) and adjusted odds ratios (AORs). The COR is computed using univariate logistic regression. All variables that were clinically important and with p <0.17 in a univariate analysis were included in the backward multivariable logistic regression model to adjust for confounding effect (AORs). The statistical significance was considered at p <0.05 for associated variables within 95% confidence interval.

### Ethical

The study was conducted in accordance with the ethical clearance obtained from Institutional Review Board of College of Health Sciences, Jimma University, Jimma, Ethiopia (Reference No. HRPGC /40192/2016) and Institutional Review Board of Wollega University, Nekemte, Ethiopia (Reference No. WU/RD/210/2010) S1 File. Official permission was also obtained from National Agency for the Immigration and Return, Regional Refugee Administrator and local Refugee Camps official to conduct the study on the refugee camps, while for the surrounding communities, official permission was obtained from the Regional Health Department and Institutional Officials through official support letter from Jimma University, Jimma, Ethiopia (Reference No. HRPGC/40191/2016). To maintain the confidentiality of patients participated in the study, the patient identifiers were coded, the data were collected by trained health professional working in the TB treatment centers, and data were kept in the secure place accessible to the researcher only.

## Result

### Socio demographic characteristics of the patients

Of the 5148 individuals on anti TB treatment in the course of the study period, 860 (20.8%) refugees and 3284 (79.2%) the SCs, totally 4144 TB patients met the inclusion criteria (Fig 1). From the total study participants female accounted 54.3% among the refuge and 41.7% in the SCs, yielding a male to female ratio of 1:1.18 and 1.39:1 among refugees and the SCs, respectively. The majority of the study participants were between the age of 15–35 with peak age between 24–35 year old (28.9%), and followed by 15–24 (24.3%) years old, with mean age of 25.12 (standard deviation 14.60) and 26.52 (standard deviation 15.0.5) for refugee and the SCs, respectively. Majority of the study participant’s category was new cases (93.1%) follows by relapse cases (4.0%) and defaulter cases (1.4%) in the previous treatment (Table 1).

**Table 1:** Socio-demographic characteristic of refugees and surrounding communities registered TB cases (n = 4144) in Gambella Regional State, South West Ethiopia, 2009–2017.

Variables	Refugee, n (%)				Surrounding Communities, n (%)				Total
	PTB+	PTB-	EPTB	Total	PTB+	PTB-	EPTB	Total	
**Residence area**									
Urban	495(57.6)	169(19.7)	196(22.8)	860(100)	486(39.1)	524(42.1)	23418.8)	1244(37.9)	2104(50.8%)
Rural	0(0.0)	0(0.0)	0(0.0)	0(0.0)	729(35.7)	946(46.4)	365(17.9)	2040(62.1)	2040(49.2%)
**Sex**									
Male	235(45.7)	75(59.8)	83(19.1)	393(45.7)	746(10.4)	829(56.4)	338(33.2)	1913(58.3)	2306(55.6%)
Female	260(55.7)	94(20.1)	113(24.2)	467(54.3)	469(34.2)	641(46.8)	261(19.0)	1371(41.7)	1838(44.4%)
**Age**									
≤14	23(11.9)	65(33.7)	105(54.4)	193(22.4)	67(10.4)	362(56.4)	213(33.2)	642(19.5)	835(20.1%)
15–24	144(70.2)	28(13.7)	33(16.1)	205(23.8)	383(24.4)	271(47.8)	147(33.8)	801(18.4)	1006(24.3%)
25–34	177(71.7)	38(15.4)	32(13.0)	247(28.7)	419(44.1)	405(42.6)	127(13.4)	951(29.0)	1198(28.9%)
35–44	81(64.8)	30(24.0)	14(11.2)	125(14.5)	201(43.6)	207(44.9)	53(11.5)	461(14.0)	586(14.1%)
45–54	52(85.2)	3(4.9)	6(9.8)	61(7.1)	101(37.8)	126(47.2)	40(15.0)	267 (8.1)	328(7.9%)
55–64	14(66.7)	5(23.8)	2(9.5)	21(2.4)	28(25.9)	67(62.0)	13(12.0)	108(3.3)	129(3.1%)
≥65	4(50.0)	0(0.0)	4(50.0)	8(0.9)	16(29.6)	32(59.3)	6(11.1)	54(1.6)	62(1.5%)
**Mean age (Standard deviation)**			25.12(14.60)		26.52(15.0.5)				
**Patient category**									
New	456(56.1)	164(20.2)	193(23.7)	813(94.5)	1081(35.5)	1386(45.5)	578(19.0)	3045(92.7)	3858(93.1%)
Relapse	15(88.2)	2(11.8)	0(0.0)	17(2.0)	100(66.7)	40(26.7)	10(6.7)	150(4.6)	167(4.0%)
Failure	2(100.0)	0(0.0)	0(0.0)	2(0.2)	8(66.7)	2(16.7)	2(16.7)	12(0.4)	14(0.3%)
Defaulter	9(100.0)	0(0.0)	0(0.0)	9(1.0)	15(31.2)	30(62.5)	3(6.2)	48(1.5)	57(1.4%)
Transfer	13(68.4)	3(15.8)	3(15.8)	19(2.2)	11(37.9)	12(41.4)	6(20.7)	29(0.9)	48(1.2%)
**HIV status**									
Positive	135(59.5)	55(24.2)	37(16.3)	227(26.4)	289(39.9)	345(47.7)	90(12.4)	724(22.0)	951(22.9%)
Negative	279(55.8)	94(18.8)	127(25.4)	500(58.1)	773(39.5)	827(42.2)	359(18.3)	1959(59.7)	2459(59.3%)
Unknown	81(60.9)	20(15.0)	32(24.1)	133(15.5)	140(24.7)	287(50.7)	139(24.6)	566(17.2)	699(16.9%)
Refused the test	0(0.0)	0(0.0)	0(0.0)	0(0.0)	13(37.1)	11(31.4)	11(31.4)	35(1.1)	35(0.8%)
**Study institution**									
Gambella Hospital	0(0.0)	0(0.0)	0(0.0)	0(0.0)	864(34.4)	1184(47.1)	467(18.6)	2515(76.6)	2515(60.7%)
Gambella HC	0(0.0)	0(0.0)	0(0.0)	0(0.0)	164(34.8)	210(44.6)	97(20.6)	471(14.3)	471(11.4%)
Bonga HC	0(0.0)	0(0.0)	0(0.0)	0(0.0)	33(57.9)	16(28.1)	8(14.0)	57(1.7)	57(1.4%)
Pungidio HC	0(0.0)	0(0.0)	0(0.0)	0(0.0)	154(63.9)	60(24.9)	27(11.2)	241(7.3)	241(5.8%)
PR- 1 HC	244(56.2)	92(21.2)	98(22.6)	434(50.5)	0(0.0)	0(0.0)	0(0.0)	0(0.0)	434(10.5%)
PR- 2 HC	223(61.3)	61(16.8)	80(22.0)	364(42.3)	0(0.0)	0(0.0)	0(0.0)	0(0.0)	364(8.8%)
JRHC	28(45.2)	16(25.8)	18(29.0)	62(7.2)	0(0.0)	0(0.0)	0(0.0)	0(0.0)	62(1.5%)
**Year of anti TB treatment initiated**									
**2009**	45(64.3	11(15.7)	14(20.0)	708.1)	139(28.5)	230(47.1)	119(24.4)	488(14.9)	558(13.5)
**2010**	54(75.0)	1(1.4)	17(23.6)	72(8.4)	162(37.7)	187(43.5)	81(18.8)	430(13.1)	502(12.1)
**2011**	55(64.7)	7(8.2)	23(27.1)	85(9.9)	128(35.6)	144(40.0)	88(24.4)	360(11.0)	445(10.7)
**2012**	46(55.4)	23(27.7)	14(16.9)	83(9.7)	149(42.1)	154(43.5)	51(14.4)	354(10.8)	437(10.5)
**2013**	56(59.6)	30(31.9)	8(8.5)	94(10.9)	160(38.0)	204(48.5)	57(13.5)	421(12.8)	515(12.4)
**2014**	58(61.1)	17(17.9)	20(21.1)	95(11.0)	149(38.8)	165(43.0)	70(18.2)	384(11.7)	479(11.6)
**2015**	68(60.7)	15(13.4)	29(25.9)	112(13.0)	180(35.5)	236(46.5)	91(17.9)	507(15.4)	619(14.9)
**2016**	113(45.4)	65(26.1)	71(28.5)	249(29.0)	148(43.5)	150(44.1)	42(12.4)	340(10.4)	589(14.2)
Total	495(57.6)	169(19.7)	196 (22.8)	860(20.8)	1215(37.0)	1470(44.8)	599(18.2)	3284(79.2)	4144(100.0)

Note: PTB+, Smear positive pulmonary tuberculosis; PTB-, smear negative pulmonary tuberculosis; EPTB, Extra pulmonary tuberculosis; JRHC, Jawi Refugee Health Center; PR1-HC, Pugnido 1 Refugee Health Center, Pugnido 2 Refugee Health Center; HC, Health Center; n (%), number (percentage). There were difference in clinical presentation (X^2^ = 185.834; P<0.0001) and trend of all form of TB (X^2^ trend = 207.720; P<0.0001) in the two study populations.

Among the patients with known HIV sero-status, 27.9% (951/3410) were HIV co-infected. The TB/HIV-co-infection rate was slightly higher among refuge 31.22% (227/727) compared to the SCs 26.98% (724/2683), (Table 1).

### Tuberculosis case profiles and its trend

The clinical record of the patients revealed that there were differences in clinical presentation of TB in the two study population (X^2^ = 185.834; P<0.0001). In the refugees, 495(57.6%), 196 (22.8%) and 169 (19.7%) were PTB+, EPTB and PTB-, respectively whiles in the SCs, the PTB- were the major ones (44.8%) followed by PTB+(37.0%) and EPTB, (18.2%). Higher percentage (59.5%) of TB/HIV co-infection was recorded among PTB+ in refugees while PTB- 345 (47.7%) was the prevalent in the SCs (Table 1).

Overall trend of all form of TB in the two populations was progressively declining from 13.5% in 2009 to 10.5% in 2012. However the trend increased to 14.2% in 2016 as depicted in Table 1. There is a significant difference in the trend of all form of TB in the two study populations (X^2^ trend = 207.720; P<0.0001). In refugees, all forms of TB were gradually increasing from 8.1% to 29.0% across the study years while in the SCs it was declining from 14.95 in 2009 to 10.8% in 2012 year, but increased to 15.4% in 2015. However, it showed a decline again to 10.4% in 2016.

**Fig 2:**
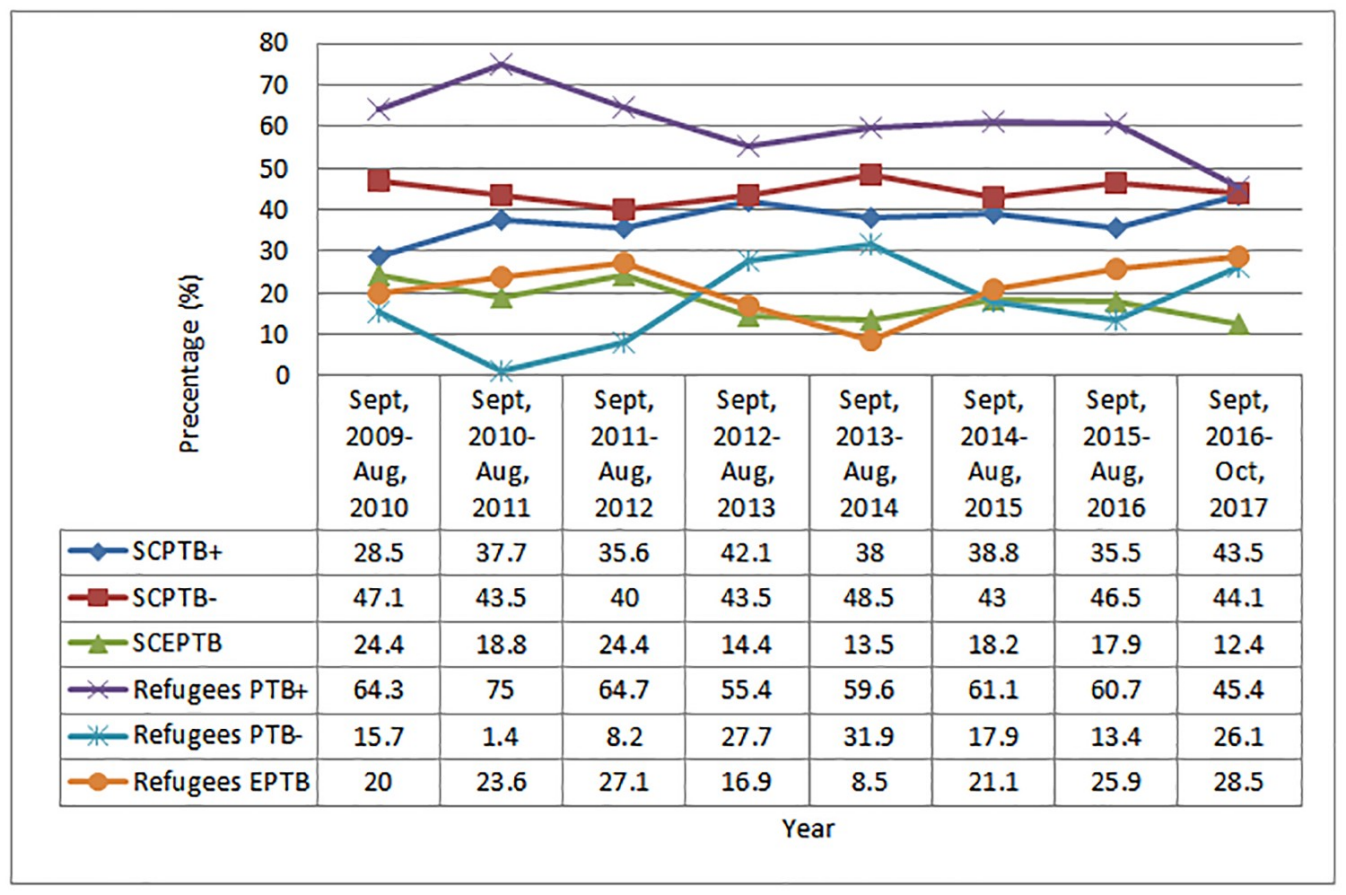
Trend of all types of registered TB cases (n = 4144) among refugees and surrounding communities in Gambella Regional State, South West Ethiopia, 2009–2017. Note: SCPTB+, Surrounding Communities smear positive pulmonary TB; SCPTB-, Surrounding communities smear negative pulmonary TB; SCEPTB, Surrounding communities extra pulmonary tuberculosis; Sept, September; Aug, August. There is significant difference in the trend of all form of TB in the two study populations (X^2^ trend = 207.720; P<0.0001).

The eight years trends of all forms of TB cases are shown in Fig 2. The trend of PTB+ in refugees was progressively declining from 64.4% in 2009 to 45.5% in 2016 year while the other forms of TB cases were increasing with small irregularity in the trend. Contrary to this, the trend of PTB+ was progressively increasing from 28.5% to 43.5% while the EPTB declined from 24.4% to 12.4% in the SCs in the course of the study period.

### Tuberculosis treatment outcomes

A total of 4144 TB patients were registered for anti TB treatment from September, 2008 to October, 2017. Of this, 1386 (33.4%), 2146 (51.8%), 196 (4.7%), 25 (0.6%), and 391(9.4%) were cured, treatment completed, dead, failure and defaulter, respectively. There was difference in the treatment outcome between the two study populations (X^2^ = 170.915; P<0.0001). The treatment success was 74.2% and 88.1% in refugees and the SCs, respectively. Out of the total 860 registered refugee TB patients, 336 (39.1%), 302 (35.1%), 56 (6.5%), 13 (1.5%), and 153 (17.8%) were cured, treatment completed, dead, failure and defaulter, respectively. A similar trend was also observed in the SCs where 1050 (32.0%), 1844 (56.1%), 140 (4.3%), 12 (0.4%), and 238 (7.2%) were cured, treatment completed, dead, failure and defaulter, respectively (Fig 1 and Table 2).

**Table 2:** Trends of TB treatment outcomes among refugees and surrounding communities registered TB cases in Gambella Regional State, South West Ethiopia, 2009–2017.

Treatment outcome	Year								Total
	Sept, 2009-Aug, 2010	Sept, 2010-Aug, 2011	Sept, 2011-Aug, 2012	Sept, 2012-Aug, 2013	Sept, 2013-Aug, 2014	Sept, 2014-Aug, 2015	Sept, 2015-Aug, 2016	Sept, 2016-Oct, 2017	
**SCs**									
Cured	121(24.8)	135(31.4)	106(29.4%)	130(36.7)	149(35.4)	128(33.3)	146(28.8)	135(39.7)	1050(32.0)
RX com	332(68.0)	229(53.3)	204(56.7)	187(52.8)	223(53.0)	188(49.0)	301(59.4)	180(52.9)	1844(56.1)
** Total**	**453(92.8)**	**364(84.7)**	**310(86.1)**	**317(89.5)**	**372(88.4)**	**316(82.3)**	**447(88.2)**	**315(92.6)**	**2894(88.1)**
Died	23(4.7)	21(4.9)	24(6.7)	14(4.0)	23(5.5)	17(4.4)	9(1.8)	9(2.6)	140(4.3)
Failure	3(0.6)	1(0.2)	1(0.3)	1(0.3)	1(0.2)	0(0.0)	3(0.6)	2(0.6)	12(0.4)
Defaulter	9(1.8)	44(10.2)	25(6.9)	22(6.2)	25(5.9)	51(13.3)	48(9.5)	14(4.1)	238(7.2)
** Total**	**35(7.2)**	**66(15.7)**	**50(14.2)**	**37(11.3)**	**49(12.2)**	**68(18.9)**	**60(12.9)**	**25(7.8)**	**390(11.9)**
**Refugees**									
Cured	34(48.6)	40(55.6)	36(42.4)	26(31.3)	38(40.4)	50(52.6)	48(42.9)	64(25.7)	336(39.1)
RX com	18(25.7)	13(18.1)	36(42.4)	41(49.4)	38(40.4)	26(27.4)	30(26.8)	100(40.2)	302(35.1)
** Total**	**52(74.3)**	**53(73.6)**	**72(84.7)**	**67(80.7)**	**76(80.9)**	**76(80.0)**	**78(69.6)**	**164(65.9)**	**638(74.2)**
Died	4(5.7)	5(6.9)	5(5.9)	8(9.6)	10(10.6)	2(2.1)	10(8.9)	12(4.8)	56(6.5)
Failure	0(0.0)	0(0.0)	0(0.0)	4(4.8)	0(0.0)	2(2.1)	2(1.8)	5(2.0)	13(1.5)
Defaulter	14(20.0)	14(19.4)	8(9.4)	4(4.8)	8(8.5)	15(15.8)	22(19.6)	68(27.3)	153(17.8)
** Total**	**18(25.7)**	**19(26.4)**	**13(15.3)**	**16(19.3)**	**18(19.1)**	**19(20.0)**	**34(30.4)**	**85(34.1)**	**222(25.8)**

Note: SCs, surrounding communities; RX com, Treatment Complete; Sept, September; Aug, August. There was significant difference in mean treatment success rate (X^2^ = 92.887; P<0.0001) in the study population.

### Trend of TB treatment outcome and its treatment success rate

The trend of TB treatment outcome for the two study populations was presented in Table 3. The overall mean treatment success rate of the TB patients (n = 4144) was 85.2% across the eight years. There was a significant difference in mean treatment success rate (X^2^ = 92.887; P<0.0001) in the study population. The mean treatment success rate was 74.2% and 88.1% for refugees and the SCs, respectively. Similarly, the treatment success rate was higher among the study population of rural residents, patients less than 35 year old, female, new cases, PTB+ form, TB/HIV-non-infected and surrounding communities (Tables 2 and 3).

**Table 3:** Factors associated with poor (unsuccessful)TB treatment outcome among refugees and surrounding communities (SCs) TB cases registered in Gambella Regional State, South West Ethiopia, 2009–2017.

Character	Total	Unsuccessful outcome	COR(95%CI)	P-value	AOR(95%CI)	P-value
**Area of residence**						
Urban	1959(49.1)	374(19.1)	1.00		1.00	
Rural	2034(50.9)	238(11.7)	**0.56(0.47–0.67)**	**0.000**	0.77(0.62–0.97)*	**0.025**
**Sex**						
Male	2216(55.5)	364(16.4)	1.00		1.00	
Female	1777(44.5)	248(14.0)	**0.82(0.69–0.98)**	**0.031**	**0.76(0.63–0.91)**	**0.003**
**Age**						
≤14	808(20.2)	106(13.1)	1.00		1.00	
15–24	981(24.6)	130(13.3)	1.01(0.77–1.33)	0.934	1.07(0.79–1.43)	0.672
25–34	1155(28.9)	184(15.9)	1.25(0.97–1.62)	0.084	1.067(0.79-.43)	0.184
35–44	559(14.0)	97(17.4)	**1.39(1.03–1.87)**	**0.031**	**1.38(1.0–1.91**)*	**0.049**
≥45	490(12.3)	95(19.4)	**1.59(1.17–2.15)**	**0.000**	1.77(1.28–2.44)*	**0.001**
**TB form**						
PTB+	1674(41.9)	265(15.8)	1.00		1.00	
PTB-	1559(39.0)	219(14.0)	0.87(.71–1.05)	0.156	1.15(0.93–1.43)	0.184
EPTB	760(19.0)	128(16.8)	1.07(0.85–1.36)	0.530	1.34(1.04–1.73)*	**0.023**
**Patient category**						
New	3714(93.0)	558(15.0)	1.00		1.00	
Retreated(R+F+D)	235 (5.9)	45(19.1)	1.34(0.96–1.87)	0.089	1.53(1.07–2.17)*	**0.018**
Transfer	44(1.1)	9(20.5)	1.45(0.69–3.04)	0.320	1.04(0.48–2.24)	0.915
**HIV status**						
Positive	924(23.1)	189(20.5)	1.00		1.00	
Negative	2355(59.0)	321(13.6)	0.61(0.50–0.75)	0.000	0.63(0.51–0.77)*	**<0.001**
Unknown	680(17.0)	100(14.7)	0.67(0.51–0.87)	0.003	0.74(0.55–1.00)	0.050
Refused the test	34(0.9)	2(5.9)	0.24(.06 1.02)	0.054	0.44(0.10–1.87)	0.271
**Study population**						
Surrounding communities	3133(78.5)	390(12.4)	1.00		1.00	
Refugee	860(21.5)	222(25.8)	**2.45(2.03–2.95)**	0.00	2.17(1.69–2.77)*	**<0.001**
**Year of treatment**						
**Sept, 2009-Aug, 2010**	556(13.9)	53(9.5)	1.00		1.00	
**Sept, 2010-Aug, 2011**	493(12.3)	85(17.2)	**1.97(1.37–2.85)**	0.000	2.11(1.44–3.11)*	**<0.001**
**Sept, 2011-Aug, 2012**	437(10.9)	63(14.4)	**1.60(1.08–2.36)**	0.018	1.56(1.04–2.34)*	**0.033**
**Sept, 2012-Aug, 2013**	410(10.3)	53(12.9)	1.42(0.94–2.11)	0.096	1.33(0.87–2.03)	0.190
**Sept, 2013-Aug, 2014**	497(12.4)	67(13.5)	**1.48(1.012.17)**	0.045	1.44(0.96–2.15)	0.078
**Sept, 2014-Aug, 2015**	455(11.4)	87(19.1)	**2.24(1.55–3.24)**	0.000	2.14(1.46–3.13)*	**<0.001**
**Sept, 2015-Aug, 2016**	576(14.4)	94(16.3)	**1.85(1.29–2.65)**	0.001	1.89(1.31–2.75)*	**0.001**
**Sept, 2016-Oct, 2017**	569(14.2)	110(19.3)	**2.27(1.60-.23)**	0.000	1.89(1.30–2.74)*	**0.001**

Note: Retreated is the sum of relapse(R), defaulter (D) and Failure (F); COR, Crude Odds Ratio; 1:00, Reference; *, statistically significant; AOR, Adjusted Odds ratio; CI, Confidence interval; Sept, September; Aug, August.

### Factors associated with unsuccessful treatment outcomes

Multivariate logistic regression revealed (Table 3), that the risk of unsuccessful TB treatment outcome was significantly higher among refugees (AOR = 2.17; 95% CI: 1.69–2.77), retreated cases (AOR = 1.53; 95% CI: 1.07–2.17), age group between 35–44 years (AOR = 1.38; 95% CI: 1.0–1.91), greater than 44 years old (AOR = 1.77; 95% CI: 1.28–2.44), and patients with EPTB form (AOR = 1.34; 95% CI: 1.04–1.73) compared to their counterparts. Moreover, the comparison of patients coming from rural area (AOR = 0.77; 95% CI: 0.62–0.97) with the urban, female (AOR = 0.76; 95% CI: 0.63–0.91) with the male and non TB/HIV co-infected (AOR = 0.63; 95% CI: 0.51–0.77) with TB/HIV co-infected ones were more likely to be successfully treated.

## Discussion

In the eight years retrospective study, 4144 TB patients with complete information on treatment outcome were extracted and included in the analysis to evaluate trend in TB form, treatment outcome and factor associated with unsuccessful outcome. Out of these, 860 and 3284 were from refugees and the SCs, respectively. The result shows presence of difference in the trend, TB form presentation and treatment outcome of TB in the two study populations. Variations were also noticed in clinical presentation and treatment outcome during the study period. These outcomes might benefit the national and regional state TB control progamme and other organizations working on the refugees to look for possible evidence based strategy to address existing difference in treatment outcome and trend of TB notification among refugees and the SCs in the country.

The global gender difference in the TB notification was also found within the two study populations [1], where most patients were male in the SCs and female in refugees, with yielding a male to female ratio of 1.39:1 and 1:1.18 among SCs and refugees, respectively. This high TB notification in male among the SCs supports previous studies in Ethiopia [14–16]. Similarly, the presence of more TB patients in female among refugees supports previous study in Pakistan [17]. This gender inequality between refugees and the SCs as well as other previous studies [14–17] could be due to differences in biological, socio-economic, cultural and access to health facilities [17–19]. The presence of narrow difference in gender ratio was attributed to an improvement in the access to TB control services in the area [14].

One-third of TB cases were noted among HIV positive refugees in this study which is higher than the SCs (26.98%). This supports the existing fact that HIV co-morbidity is one of the most important risk factors for TB [20]. The presence of high (53.2%) TB notification among productive age groups [15–34] supports the global report of TB-associated morbidity and mortality that occurs mainly in the economically productive age group [1]. This could create a challenge for the socio-economic development of the community, and the nation at large.

The occurrences of a higher PTB+ in refugees and PTB- in the SCs found in this study could be attributed to the differences in socioeconomic status [21], living conditions [22], and prevalence of HIV/AIDS [14] between the two population as refugees were more likely to be nutritionally poor, live in crowded conditions [23] and have high (31.22%) TB/HIV co-infection.

The study also revealed a gradual increase in all forms of TB in refugees that contrast with the trend in the SCs in the present and other studies [14–16, 20]. This is attributed to the increasing number of refugee population in the study refugee camps following political instability in South Sudan since 2013 [13] and degradation of health care system in the home country that imposes a negative impact on TB diagnosis, treatment and prevention efforts.

The overall mean treatment success rate of the TB patients was 85.2% across the eight years and there was also significant difference in mean treatment success rate in both study populations. The study depicted that 39.1% and 35.1% of registered refugee patients under TB control program were cured and treatment complete, that accounted for overall mean treatment success rate of 74.2%. This is consistent with treatment success rate among refugees in different part of the world, ranging from 66.5% to 77.5% [24–27]. However, this treatment outcome rate is lower than the global target (>90%) [1], the treatment success rate among the SCs in the present study (88.1%), the last WHO report of nation treatment success rate among new case registered (84.0%) [1] and other studies [1, 14, 15, 20]. This observed low treatment success rate in refugees was attributed to the presence of higher death (6.5%) and defaulter rate (17.8%) among the population of the study. Moreover, the refugee population might have problems of malnutrition and other coexisting illnesses such as HIV co- infection (31.22%) as found in our study that may lead to excessively high death rates, and thus low cure rates.

The treatment success rate in the SCs in this study was similar to treatment success rate reported from Dabat (87.8%) [28] and Tigray (89.2%) in northern Ethiopia [29]. However, this treatment success rate among the SCs study population was lower than the TB success rate in Northwest Ethiopia (91.4%) [30]. In addition, the treatment success rate found in SCs in this study was higher than reports from different geographical areas in Ethiopia, from where different treatment success rates ranging from 19% to 85% were reported [15, 16, 31, 32]. The successful treatment outcome in the SCs in this study justifies the contribution of institution DOTs performance in TB control program in the study area.

Understanding factors associated with unsuccessful treatment outcomes can help to design appropriate evidence based intervention to reduce morbidity and mortality. The multivariate logistic regression revealed that the risk of unsuccessful treatment outcomes was significantly higher among refugees (AOR = 2.17; 95% CI: 1.69–2.77) compared to the SCs in present study. The finding supports the existing facts [24]. The observed difference in this study was mainly attributed to the presence of high defaulter rate among refugees (17.8%), which is consistent with the existing literatures that associates unsuccessful treatment outcomes with inadequate treatment adherence. In addition, patients’ behaviors and attitudes towards the disease were major factors that could also affect treatment adherence [33].

As expected, the retreated cases were associated with unsuccessful treatment outcome compared to new cases. Patients with previous treated cases have high level of treatment failure as result of possible development of drug resistance in the repeated exposure [34]. In addition, patient-related behavior might contribute more in marginalized communities like refugees as previous studies showed. Patients who had lost to follow-up history could be reluctant and tend to interrupt their treatment again [31, 35]. Age was also found to be a predicator factors for unsuccessful treatment outcomes in the study, where the risk of unsuccessful TB treatment outcome increased with increasing age of the TB patients aged above 35 years. This is supported by other studies [31, 36, 37].

Supporting the recent report from Sidama Zone, Ethiopia [14], patients from rural areas had significantly unsuccessful treatment outcome compared to patients from urban areas. This could be explained by the presence of more adverse conditions such as living in highly overcrowded areas [38]; poor socioeconomic status [39, 40] and a high prevalence of HIV/AIDS [41] in patients coming from rural area.

The study also found that there were higher odds unsuccessful treatment outcome among EPTB compared with PTB+. This confirms the findings of other study [15]. The finding might be attributed to the absence of treatment prognostic test for EPTB, where patients’ treatment response was managed only with clinical based unlike PTB+ that have sputum test. In PTB+, patients’ response was monitored at 2nd, 5th, and 7th months using sputum tests in addition to clinical progression of the patients. Sputum tests have immense contribution to know the patients’ treatment response on time and manage the condition to prevent further morbidity and development of drug resistance.

Interestingly, the present study revealed that female (AOR = 0.76; 95% CI: 0.63–0.91) were less likely to be unsuccessfully treated (to default, to fail, and die) compared to male. This was consistent with a study conducted in southern [42] and Northwestern Ethiopia [43]. The lower social interaction outside home, mobility from place to place and other related TB risk behaviors of females might contribute to this lower death, failure and default among the female. There was also less treatment unsuccessful outcome among non TB/HIV co-infected cases (AOR = 0.63; 95% CI: 0.51–0.77) compared to TB/HIV co-infected ones. The finding is consistent with other studies [31, 32]. This partially justifies the impact of drug-drug interaction and overlapping drug toxicities between antiretroviral therapy with anti-TB, and impaired immunity by HIV/AIDS on treatment response in the TB/HIV co-infected cases.

The findings of this study should be seen in light of the fact that the study incorporated data of patients both from refugees and surrounding communities with completed information of their treatment outcome in the last eight years. The study excludes patients transferred to other health facilities. In addition, important potential patient information that could affect TB treatment outcome were not obtained because of retrospective nature of the study such as distance from the treatment center, knowledge, health seeking behaviors, compliance with treatment, drug resistance status, medication side effect, occupation, educational level and other co-morbidity with chronic illnesses. Hence, these limitations of the study need to be considered while interpreting the findings.

## Conclusion

The study confirmed that there was low treatment success rate among refugees compared to the SCs (88.1%) and the global target (>90%). There was a difference in the trend, TB form presentation and treatment outcome of TB across the study period in the two study populations. The trend of all forms of TB was steadily increasing among refugees while declining in the SCs. Being refugee, retreated case, patient’s age ≥35 years old, EPTB form, gender, rural patient address and HIV status were predictor factors for unsuccessful treatment outcome. Hence, the study urges the need for strengthened TB prevention program among refugees with consideration of identified predicator factors to prevent the potential effect of hosting refugee to the SCs and the nations at large.

## Supporting information

S1 File Letter of ethical approval from two institutions. (PDF)

S1 Table Data extraction template for factors associated with unsuccessful treatment outcome in tuberculosis patients among refugees and their surrounding communities in Gambella Regional State, Ethiopia project, 2017. (DOCX)
