# Supplementary material for: Factors associated with unsuccessful treatment outcome in tuberculosis patients among refugees and their surrounding communities in Gambella Regional State, Ethiopia
Source: PLoS One. 2018 Oct 18;13(10):e0205468. doi: 10.1371/journal.pone.0205468 (PMC6193657; doi:10.1371/journal.pone.0205468)
Supplement: S1 File — (PDF) [file pone.0205468.s001.pdf]

JIMMA UNIVERSITY

ጅማ ዩኒቨርሲቲ

ቁጥር
Ref.No. HRPGC/40192/2016

ቀን
Date 10/03/2016

Institutional Review Board (IRB),
College of Health Sciences,
JU, Jimma
Tel: +251471120945
E-mail: mirkuzie.woldie@ju.edu.et

To Mr. Eyasu Ejeta

Subject: Ethical approval of your research protocol

The IRB of College of Health Sciences has reviewed your mega research project entitled: ***"Molecular epidemiology and drug resistance of Mycobacterium tuberculosis among refuges and its impact on the surrounding communities in Ethiopia."*** This is to notify that this research protocol as presented to the IRB meets the ethical and scientific standards outlined in national and international guidelines. Hence, we are pleased to inform you that your protocol is *ethically cleared.*

We strongly recommend that any significant deviation from the methodological details indicated in the approved protocol must be communicated to the IRB before they are implemented.

With regards,

Mirkuzie Woldie (MD,MPH)
Research & Post Graduate
Coordinator

Jimma University
College of Health Science
Research & Postgraduate Coordinating Office

ወለጋዩኒቨርሲቲ
Wollega University
Research Ethics Review Committee (RERC)

ጥር/Ref.No WU, RD, 210, 2010
ቀን/Date 24, 09, 2010

To: Prof/Dr./**Mr.**/Mrs. **Eyasu Ejeta**

From: Research Ethics Review Committee

WU

Subject: Issuing Ethical Clearance Certificate

The Research Ethics Review Committee (RERC) of Wollega University in its meeting held on Date 20/5/2018, (Minutes No.:05/2010) has examined your research proposal entitled **"Magnitude of tuberculosis case notification and factor associated with poor treatment outcomes among immigrant refugees and surrounding communities in Gambella Regional State, Ethiopia".** The Proposal is approved for implementation and has been certified and given this ethical clearance to conduct the proposed research College of Health Sciences.

This certificate will serve for one year (from 20/5/2018 to 18/5/2019)

Name: **Diriba Diba(PhD)**
Chair Person
Signature: [signature]
የምርምርና ኢኖቬሽን ዳይሬክተር
Research and Innovation Director
Date:

Name: **Worku Dechassa(MPH)**
Secretary
signature: [signature]
Date: 24/09/2010

የምርምር፣ ስርፀትና ምክር አገልግሎት
Wollega University
RESEARCH EXTENSION & CONSULTANCY SERVICES

CC
Research and Tech. Transfer Vice President Office
Research and Innovation Directorate office
Institutions/Centers/Departments
College Research Associate Dean
WU
