# Supplementary material for: Factors associated with unsuccessful treatment outcome in tuberculosis patients among refugees and their surrounding communities in Gambella Regional State, Ethiopia
Source: PLoS One. 2018 Oct 18;13(10):e0205468. doi: 10.1371/journal.pone.0205468 (PMC6193657; doi:10.1371/journal.pone.0205468)
Supplement: S1 Table — (DOCX) [file pone.0205468.s002.docx]

**S1 Table. Data extraction** **template for factors associated with unsuccessful treatment outcome in tuberculosis patients among refugees and their surrounding communities in Gambella Regional State, Ethiopia project, 2017**

| **Patient Code** | **Patient Residence** | **Age** | **Sex** | **Patient category** | **TB form** | **HIV status** | **Rx outcome** | **Year of Treatment** | **Treatment Centers** | **Study population** |
| --- | --- | --- | --- | --- | --- | --- | --- | --- | --- | --- |
|  |  |  |  |  |  |  |  |  |  |  |
|  |  |  |  |  |  |  |  |  |  |  |
|  |  |  |  |  |  |  |  |  |  |  |
|  |  |  |  |  |  |  |  |  |  |  |
|  |  |  |  |  |  |  |  |  |  |  |
|  |  |  |  |  |  |  |  |  |  |  |
|  |  |  |  |  |  |  |  |  |  |  |
|  |  |  |  |  |  |  |  |  |  |  |
|  |  |  |  |  |  |  |  |  |  |  |
|  |  |  |  |  |  |  |  |  |  |  |
|  |  |  |  |  |  |  |  |  |  |  |
|  |  |  |  |  |  |  |  |  |  |  |
|  |  |  |  |  |  |  |  |  |  |  |
|  |  |  |  |  |  |  |  |  |  |  |
|  |  |  |  |  |  |  |  |  |  |  |
|  |  |  |  |  |  |  |  |  |  |  |
|  |  |  |  |  |  |  |  |  |  |  |
|  |  |  |  |  |  |  |  |  |  |  |
|  |  |  |  |  |  |  |  |  |  |  |
|  |  |  |  |  |  |  |  |  |  |  |
|  |  |  |  |  |  |  |  |  |  |  |
|  |  |  |  |  |  |  |  |  |  |  |
|  |  |  |  |  |  |  |  |  |  |  |
|  |  |  |  |  |  |  |  |  |  |  |
|  |  |  |  |  |  |  |  |  |  |  |
|  |  |  |  |  |  |  |  |  |  |  |
|  |  |  |  |  |  |  |  |  |  |  |
|  |  |  |  |  |  |  |  |  |  |  |
|  |  |  |  |  |  |  |  |  |  |  |
|  |  |  |  |  |  |  |  |  |  |  |
|  |  |  |  |  |  |  |  |  |  |  |
|  |  |  |  |  |  |  |  |  |  |  |
|  |  |  |  |  |  |  |  |  |  |  |
|  |  |  |  |  |  |  |  |  |  |  |
|  |  |  |  |  |  |  |  |  |  |  |

**Definition of variable code and value for data extraction** **template**

| **Variable** | **Code** | **Value** |
| --- | --- | --- |
| Address | 1 | Urban |
|  | 2 | Rural |
|  | 3 | Unrecorded |
| Sex | 1 | Male |
|  | 2 | Female |
| Age | Continues data |  |
| Patient category | 1 | New |
|  | 2 | Relapse |
|  | 3 | Failure |
|  | 4 | Defaulter |
|  | 5 | Transferred out |
|  | 6 | Unrecorded |
| TB form | 1 | Smear positive PTB |
|  | 2 | Smear negative PTB |
|  | 3 | EPTB |
|  | 4 | Unrecorded |
| HIV status | 1 | Positive |
|  | 2 | Negative |
|  | 3 | Unknown |
|  | 4 | Refused test |
| Rx out come | 1 | Cured |
|  | 2 | Complete |
|  | 3 | Died |
|  | 4 | Failure |
|  | 5 | Defaulter |
|  | 6 | Transferred out |
|  | 7 | Unrecorded |
| Year of treatment | Continues data (year) |  |
| DOTS/treatment center | 1 | Gambella Hospital |
|  | 2 | Gambella Health Center |
|  | 3 | Bonga Health Center |
|  | 4 | Pungidio Town Health Center |
|  | 5 | Pungidio Refugee I Health Center |
|  | 6 | Pungidio Refugee II Health Center |
|  | 7 | Jawi refugee Health Center |
| Study population | 1 | Surrounding population |
|  | 2 | Refugee |
